# Airway anomalies in flexible bronchoscopy for pediatric chronic cough: predictive value of specific clinical symptoms

**DOI:** 10.1007/s00431-025-06264-9

**Published:** 2025-07-09

**Authors:** Adem Yaşar, Özge Yilmaz, Aynur Hasanova, Berna Cansu Aritaş, Hasan Yüksel

**Affiliations:** 1 School of Medicine, Department of Pediatric Allergy and Immunology, Manisa Celal Bayar University, Manisa, Türkiye; 2 School of Medicine, Department of Pediatrics, Manisa Celal Bayar University, Manisa, Türkiye; 3 School of Medicine, Department of Pediatric Allergy and Immunology, and Pediatric Pulmonology, Manisa Celal Bayar University, Manisa, Türkiye

**Keywords:** Chronic cough, Flexible bronchoscopy, Pediatric pulmonology, Airway abnormalities, Diagnostic utility

## Abstract

Chronic cough, defined as cough lasting more than four weeks in children, is a frequent reason for pediatric pulmonology referrals. The underlying etiology often remains unclear despite extensive non-invasive evaluations, thus necessitating flexible bronchoscopy. In this study, we aimed to evaluate if specific clinical symptoms were predictive of anatomic or functional airway abnormalities detected in bronchoscopy. We analyzed 168 pediatric patients (0–18 years) who underwent flexible bronchoscopy for chronic cough at a tertiary center for this retrospective study. Demographic and clinical data, including cough characteristics and triggering factors, were extracted from medical records. Bronchoscopic findings, including structural and functional airway abnormalities, were documented. Logistic regression and receiver operating characteristic (ROC) analysis were performed to identify predictors of anatomical abnormalities. Bronchoscopy showed that 30% of the cases had unusual structures, with airway malacia (33%), vascular compression (21%), and subglottic stenosis (17%) being the most frequent issues. Younger age (OR = 0.84; 95% CI: 0.75–0.94; *p* = 0.002) and dry cough phenotype (*p* = 0.002) were significantly associated with anatomical abnormalities, whereas second-hand smoke exposure and gastroesophageal reflux disease (GERD) were not. ROC analysis demonstrated moderate predictive power (AUC = 0.74) for identifying anatomical abnormalities based on clinical parameters.

*Conclusion*: Airway malacia, vascular compression and subglottic stenosis are the most common structural and functional airway abnormalities in children with chronic cough. This study has shown that detecting anatomical or functional airway abnormalities with bronchoscopy is more common in young children with prolonged cough.

**Table:** 

**What is Known:** • *Flexible bronchoscopy is a key diagnostic tool for pediatric chronic cough when non-invasive evaluations are inconclusive, routinely identifying anomalies such as airway malacia, vascular compression, and subglottic stenosis.* • *Current guidelines offer limited guidance on which clinical features should prompt early bronchoscopy referral in children with chronic cough.*	
**What is New:** • *Younger age and a dry cough phenotype are independent predictors of anatomical airway abnormalities on flexible bronchoscopy (OR = 0.84; AUC = 0.74).* •* A symptom-based decision algorithm incorporating age, cough type, and gender demonstrates moderate discriminative power to guide bronchoscopy referral and may optimize diagnostic yield.*

**What is Known:**

• *Flexible bronchoscopy is a key diagnostic tool for pediatric chronic cough when non-invasive evaluations are inconclusive, routinely identifying anomalies such as airway malacia, vascular compression, and subglottic stenosis.*

• *Current guidelines offer limited guidance on which clinical features should prompt early bronchoscopy referral in children with chronic cough.*

**What is New:**

• *Younger age and a dry cough phenotype are independent predictors of anatomical airway abnormalities on flexible bronchoscopy (OR = 0.84; AUC = 0.74).*

•* A symptom-based decision algorithm incorporating age, cough type, and gender demonstrates moderate discriminative power to guide bronchoscopy referral and may optimize diagnostic yield.*

## Introduction

Chronic cough, defined as a cough lasting more than four weeks in children, is a prevalent symptom leading to pediatric pulmonology referrals [1]. It may lead to considerable distress for both patients and caregivers. Chronic cough often requires a more extensive evaluation to determine underlying causes, unlike acute cough, which is typically self-limiting and associated with viral infections [2].

The etiology of chronic cough in pediatric patients is multifaceted, encompassing conditions such as asthma, gastroesophageal reflux disease (GERD), protracted bacterial bronchitis, and postnasal drip associated with upper respiratory infections [3]. Also, less common but important causes like congenital airway problems, cystic fibrosis, and interstitial lung diseases should be looked at, especially when standard treatments don’t work [4]. Given this wide differential diagnosis, a systematic and evidence-based approach is critical to optimizing clinical outcomes and minimizing unnecessary interventions [5].

The initial assessment of a child presenting with chronic cough involves a comprehensive medical history, thorough physical examination, and non-invasive investigations like chest radiography and spirometry [6]. These evaluations help to distinguish between common and uncommon causes and identify red flags suggestive of serious underlying pathology. However, when these standard tests fail to clarify the etiology, the use of more advanced diagnostic methods become necessary; highlighting the critical role of flexible bronchoscopy (FB) in further assessment [7].

Flexible bronchoscopy is a well-established tool that allows for direct visualization of the airway, facilitating the detection of structural anomalies such as tracheomalacia, bronchomalacia, and airway stenosis, as well as functional abnormalities including mucosal inflammation and excessive secretions [8]. Additionally, FB enables targeted sampling for microbiological, cytological, and histopathological analyses, providing valuable insights into chronic infection, inflammation, and airway remodelling [9].

Flexible bronchoscopy has been shown to yield critical diagnostic insights in children with chronic cough, informing clinical decision-making and reducing prolonged diagnostic uncertainty [10, 11]. Even though FB is a valuable diagnostic tool, the relationship between specific clinical symptoms and bronchoscopic findings is not completely understood. Further research is needed to optimise indications for FB and enhance its clinical utility in patient care [12]. By improving our understanding of these associations, we can enhance diagnostic accuracy and optimize patient-centered management strategies [13].

Current recommendations are valuable but offer limited guidance on which young patients with chronic cough should be referred for bronchoscopy, leading to uncertainty in clinical decision-making. Especially the association between specific clinical symptoms and bronchoscopic results remains poorly defined [6, 14].

In this study, we aimed to evaluate if specific clinical symptoms were predictive of anatomic or functional airway abnormalities detected in bronchoscopy. By analyzing the association between specific clinical presentations with both structural and functional airway abnormalities, we seek to enhance the diagnostic approach and contribute to more targeted bronchoscopic evaluation strategies for this patient population.

## Methods

### Study design, setting, and ethics approval

This retrospective study was conducted at Manisa Celal Bayar University, School of Medicine, Department of Pediatric Allergy and Immunology, and Pediatric Pulmonology. The medical records of pediatric patients aged 0–18 years who underwent flexible bronchoscopy between December 29, 2017, and June 7, 2024, were reviewed.

This retrospective study was conducted in accordance with the ethical standards of the institutional research committee and with the 1964 Helsinki Declaration and its later amendments or comparable ethical standards. The protocol was approved by the Ethics Committee of Manisa Celal Bayar University School of Medicine (Approval Number: 23. 10.2024/20.478.486/2655). The requirement for informed consent was waived by the Ethics Committee due to the retrospective nature of the study.

### Study population

During the study period, a total of 522 FB procedures were performed. Among these, patients who underwent FB due to chronic cough were identified, and 168 cases met the inclusion criteria. Patients who underwent FB for other indications, such as cystic fibrosis, primary ciliary dyskinesia (PCD), and foreign body aspiration, were excluded from the study.

### Data collection

Relevant clinical data were retrospectively extracted from the patients’ medical records. The demographic and clinical characteristics of the patients, including age at presentation, gender, duration of cough, and cough characteristics (dry, productive, or metallic), were recorded. Additionally, potential triggering factors, such as sleep, eating, supine position, exertion, and nasal congestion, were evaluated. Bronchoscopy findings, including structural and functional airway abnormalities such as airway malacia, vascular compression and subglottic stenosis, were obtained from procedural reports.

### Flexible bronchoscopy procedure

Flexible bronchoscopy was performed using Fujinon EB-470S (4.9 mm external diameter, 2-mm internal diameter) or Fujinon EB-470P (3.8-mm external diameter, 1.2-mm internal diameter), depending on the patient’s age and body weight. The procedure was conducted under general anesthesia, administered via mask inhalation of nitrous oxide (NO_₂_) and intravenous (IV) propofol. Neuromuscular blockers were used in selected cases.

### Statistical analysis

Data were analyzed using Jamovi statistical software (version 2.3; The Jamovi Project, 2022). Continuous variables were presented as mean ± standard deviation (SD), and categorical variables as frequency (percentage). One-way ANOVA was used for comparisons among multiple groups, while Student’s *t*-test was applied for two-group comparisons of normally distributed continuous variables. A chi-square test or Fisher’s exact test was used for categorical variables. A logistic regression analysis was done to examine the relationship between clinical characteristics and anatomical abnormalities, showing results as odds ratios (OR) and 95% confidence intervals (CI). ROC curve analysis was performed to evaluate the predictive power of clinical parameters. Statistical significance was set at *p* < 0.05.

## Results

### Sociodemographic characteristics

Of the 168 pediatric patients undergoing flexible bronchoscopy for chronic cough, 54% (*n* = 91) were male, with a mean age of 5.9 ± 4.45 years. The mean cough duration was 9.9 ± 5.23 weeks, and the most common cough type was dry cough (63%). The most frequently reported triggering factor was nighttime sleep (31%), followed by nasal congestion (27%). Exposure to secondhand smoke was reported in 30% of patients. Among patients who underwent pulmonary function testing, no significant abnormalities were detected, whereas aeroallergen sensitivity was identified in 45% of cases (Table 1).

**Table 1:** Sociodemographic and clinical characteristics of pediatric patients undergoing flexible bronchoscopy for chronic cough

		***n*** = 168
Age (years)		5.9 (4.45)
Sex (male)		91 (54.1)
Cough duration (weeks)		9.92 (9.00)
Cough phenotype	Productive	51 (31)
	Dry	103 (62)
	Metallic	11 (7)
Triggering factor	Sleeping	54 (32)
	Eating	11 (6.5)
	Supine position	33 (19.5)
	Exercises	25 (15)
	Nasal congestion	45 (27)
Secondhand smoke (yes)		49 (29)
Skin prick test (positive)		75 (45)

()-%

### Bronchoscopy findings

**Fig. 1:**
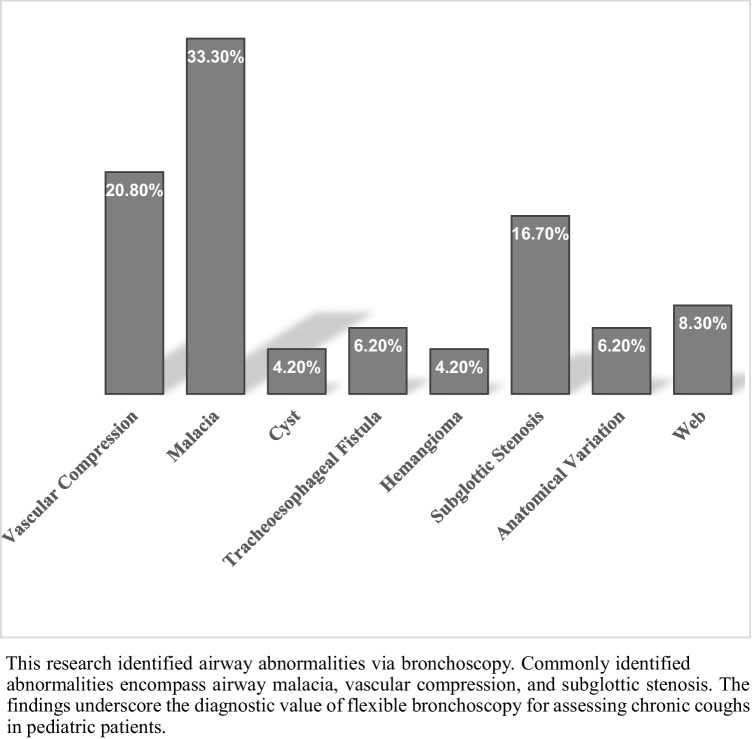
Bronchoscopic findings in pediatric patients with chronic cough

Bronchoscopy revealed anatomical and functional abnormalities in 30% (*n* = 48) of cases. The most frequently observed anomalies included airway malacia (*n* = 16), vascular compression (*n* = 10), and subglottic stenosis (*n* = 8) (Fig. 1). Among these, airway malacia was the most common finding and was predominantly observed in younger children. Vascular compression was frequently associated with stridor and respiratory distress, while subglottic stenosis was more prevalent in patients with a history of prolonged cough and recurrent respiratory infections. Patients with airway abnormalities were significantly younger than those without anomalies (4.04 ± 3.18 years, *p* = 0.001). However, cough duration did not differ significantly between these groups (*p* = 0.1). Airway abnormalities were significantly associated with male gender (*p* = 0.016) and the dry cough phenotype (*p* = 0.002). In contrast, no significant associations were found regarding secondhand smoke exposure (*p* = 0.45), aeroallergen sensitivity (*p* = 0.28), GERD history (*p* = 0.15), or the presence of protracted bacterial bronchitis (*p* = 0.55) (Table 2).

**Table 2:** Comparison of clinical and demographic factors between patients with and without anatomical airway abnormalities

		Present (***n*** = 48)	Absent (***n*** = 120)	95% CI		OR	***p*** =
Age*		4.04 ± 3.2	6.6 ± 4.7	1.16	4.06		0.001
Sex**	Male	33 (36.3)	58 (63.7)	0.21	0.86	0.42	0.01
	Female	15 (19.5)	62 (80.5)				
Cough ***	Productive	9 (16.7)	45 (83.3)				0.05
	Dry	37 (35.9)	66 (64.1)				
	Metallic	2 (18.2)	9 (81.8)				
Cough duration*	Week	8.9 ± 3.9	10.3 ± 5.6	−0.28	3.22		0.10
Triggering factors***	Sleeping	11 (20.8)	42 (79.2)				0.34
	Eating	2 (18.2)	9 (81.8)				
	Supine position	17 (51.5)	16 (48.5)				
	Exercises	2 (8)	23 (92)				
	Nasal congestion	15 (33.3)	30 (66.7)				
Skin prick test**	Present	18 (24)	57 (76)				0.28
	Absent	26 (34.7)	49 (65.3)				
Secondhand smoke**	Present	12(24.5)	37 (75.5)	0.35	1.60	0.78	0.45
	Absent	36 (30.3)	83 (69.7)				
GERD**	Present	10 (20.4)	39 (79.6)				0.15
	Absent	38 (31.9)	81 (68.1)	0.24	1.21	0.54	
PBB**	Present	3 (7.9)	35 (92.1)	0.12	0.55	0.16	0.55
	Absent	10 (12.1)	120(87.9)				

^*^Student’s *t* test

^**^ Chi square test

^***^ One way Anova

() %

*GERD* Gastroesophageal reflux disease, *PBB* Protracted bacterial bronchitis

### Prediction of anatomical abnormalities

To assess the predictive factors for anatomical abnormalities, a logistic regression analysis was performed, including age, cough duration, cough phenotype, secondhand smoke exposure, and GERD history as independent variables. Younger age was the strongest independent predictor of anatomical abnormalities (*p* = 0.002, OR = 0.84), while GERD history was associated with a lower likelihood of airway anomalies (*p* = 0.034, OR = 0.39). Neither cough duration (*p* = 0.87) nor secondhand smoke exposure (*p* = 0.48) were significant predictors.

### ROC analysis for predicting anatomical abnormalities

**Fig. 2:**
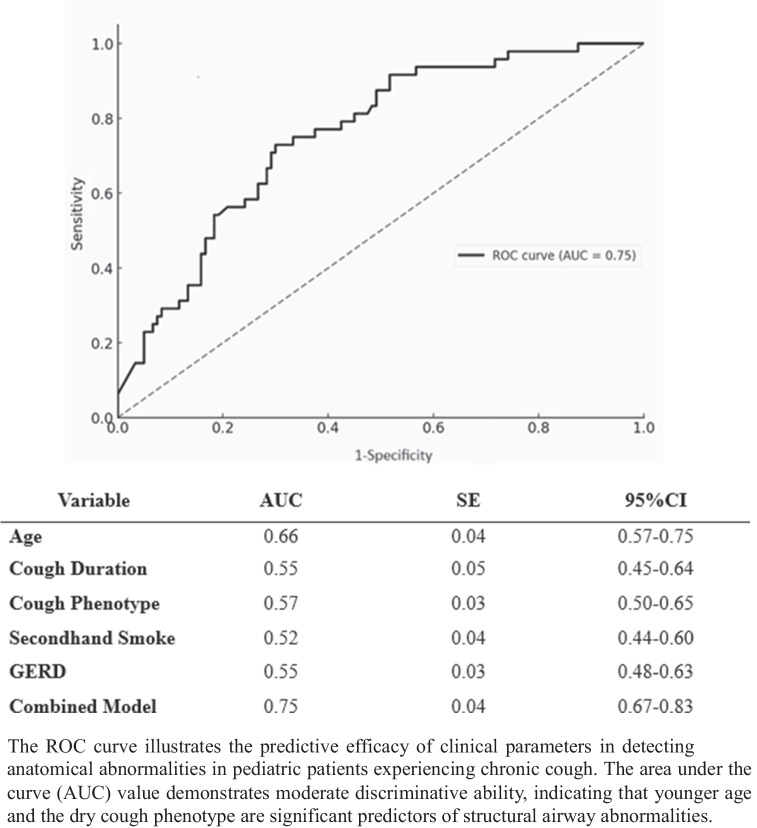
Receiver Operating Characteristic (ROC) curve analysis for predicting anatomical abnormalities

We performed a Receiver Operating Characteristic (ROC) curve analysis to assess the model’s ability to predict anatomical abnormalities. The AUC was 0.74, indicating moderate discriminative power. The optimal cutoff value was 0.34, determined using Youden’s Index. At this threshold, the model achieved 72.9% sensitivity and 70.0% specificity, balancing the ability to detect true positive cases while minimizing false positives. Scenario-based risk estimation showed that younger children (≤ 2 years) with prolonged cough (> 12 weeks) had the highest predicted risk (55.5%), while older children (> 8 years) had a lower probability (31.1%). The dry cough phenotype was also linked to a higher likelihood of anatomical abnormalities. These findings suggest that clinicians should consider early bronchoscopy evaluation in younger children with persistent dry cough, especially when no other clear cause is identified (Fig. 2).

## Discussion

This study has shown that detecting anatomical or functional airway abnormalities with bronchoscopy is more common in young children with prolonged cough. The most commonly detected abnormalities were airway malacia, vascular compression, and subglottic stenosis. Flexible bronchoscopy is an essential component in evaluating chronic cough in pediatric patients, and these results emphasize the need to use its diagnostic utility earlier in younger children.

We found airway malacia, vascular compression, and subglottic stenosis in a significant proportion of the cases in which we performed a flexible bronchoscopy for chronic cough. This emphasizes the importance of FB in cases where conventional diagnostic methods fail to determine the underlying etiology. Previous studies have reported similar rates of structural abnormalities in children undergoing bronchoscopy for chronic respiratory symptoms and support our findings [15, 16]. Our results highlighted the link between being young and having airway problems, which aligns with what we know about how birth or growth issues can significantly affect chronic cough in young children [17].

Flexible bronchoscopy remains a cornerstone in the diagnostic approach to chronic cough because it provides a direct visualization of the airway that cannot be assessed by non-invasive diagnostic methods such as spirometry or radiographic imaging [18]. The ability to assess dynamic airway changes, identify subtle mucosal abnormalities, and perform bronchoalveolar lavage (BAL) for microbiological and cytological evaluation makes FB an indispensable tool in pediatric pulmonology [19, 20]. In accordance with this, one-third of patients in our study had previously undiagnosed structural abnormalities.

The significant correlation between structural abnormalities in the airway and the male gender, as well as the presence of dry coughs in our population, highlights the need for individualized assessment strategies in diagnosis. This result is consistent with previous research suggesting that tracheomalacia and bronchomalacia are more frequently diagnosed in male infants and young children [21, 22]. Furthermore, the lack of a significant relationship between secondhand smoke exposure, aeroallergen sensitivity, and gastroesophageal reflux disease with anatomical findings suggests that while these factors may contribute to chronic cough, they do not appear to directly influence the presence of structural abnormalities [23]. This highlights the need for a direct airway check using bronchoscopy when a cough continues even without other factors like smoke exposure and allergies [24].

**Fig. 3:**
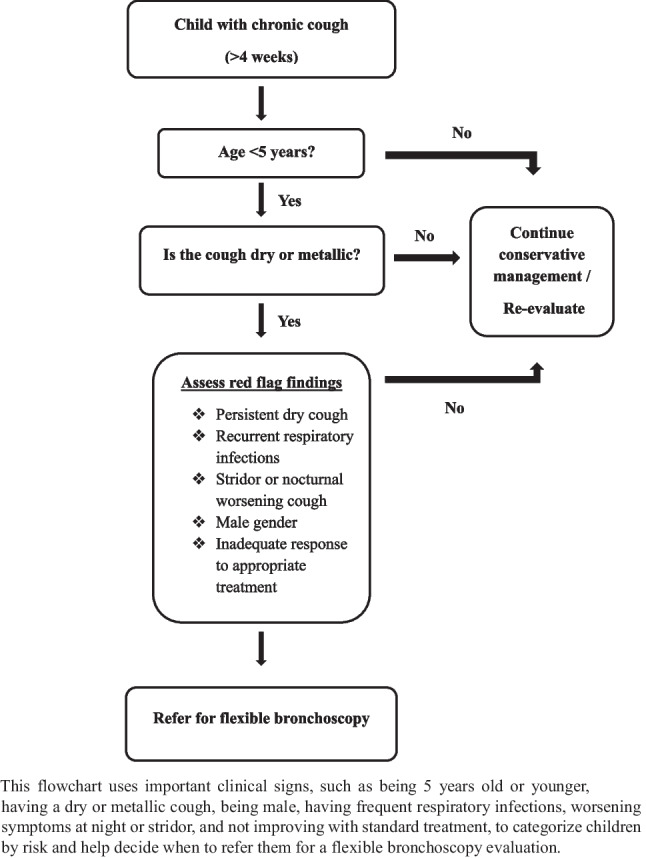
Clinical decision algorithm for bronchoscopy referral in pediatric chronic cough (study-based)

Based on our findings, we propose a decision tree for bronchoscopy referral guided by symptom profiles and clinical risk factors (Fig. 3). This algorithm includes important factors such as being under 5 years of age, having a dry or metallic cough, being male, having frequent respiratory infections, having stridor or worsening symptoms at night, and having an inadequate response to appropriate medical treatment. Including these clinical characteristics in a structured way provides a practical tool for pediatricians performing the initial assessment and following the patient to stratify risk and prioritize bronchoscopy referrals more efficiently. Implementation of such an approach in clinical practice may improve diagnostic yields, shorten diagnosis times, and prevent unnecessary investigations and treatment approaches for low-risk patients.

This study has several limitations. First, its retrospective design inherently limits causal inferences and is subject to potential selection bias. Second, as a single-center study, the generalizability of our findings to broader pediatric populations may be limited. Future multicenter prospective studies with extended follow-up periods are needed to validate these findings and provide stronger evidence for the role of FB in the diagnostic algorithm of pediatric chronic cough.

In conclusion, younger age and persistent dry cough are important predictors of functional and anatomical airway abnormalities detected in flexible bronchoscopy. Therefore, earlier consideration for bronchoscopy in these children may facilitate targeted management strategies, ultimately improving clinical outcomes and reducing diagnostic uncertainty.

## Data Availability

No datasets were generated or analysed during the current study.

## References

[1] Boonjindasup W, Thomas RJ, Yuen W, McElrea MS (2024) Role of spirometry, radiology, and flexible bronchoscopy in assessing chronic cough in children. J Clin Med 13:5720. 10.3390/jcm13195720 39407780 10.3390/jcm13195720 PMC11476545

[2] Elniny AM, Abdel Razik AM, Abo-Elezz AA, Elmeazawy R, Youssef A, Hussein MM (2024) The role of flexible bronchoscope in the evaluation of chronic cough with and without wheeze in children. Egypt J Bronchol 18:63. 10.1186/s43168-024-00317-7

[3] Chang AB, Oppenheimer JJ, Weinberger M, Rubin BK, Irwin RS (2020) Managing chronic cough as a symptom in children and management algorithms: CHEST guideline and expert panel report. Chest 158:303–329. 10.1016/j.chest.2020.01.042 32179109 10.1016/j.chest.2020.01.042

[4] Kantar A, Chang AB, Shields MD, Marchant JM, Grimwood K, Masters IB et al (2017) ERS statement on protracted bacterial bronchitis in children. Eur Respir J 50:1602139. 10.1183/13993003.02139-2016 28838975 10.1183/13993003.02139-2016

[5] Marchant JM, Masters IB, Taylor SM, Cox NC, Seymour GJ, Chang AB (2006) Evaluation and outcome of young children with chronic cough. Chest 129:1132–1141. 10.1378/chest.129.5.1132 16685002 10.1378/chest.129.5.1132

[6] Goussard P, Pohunek P, Eber E, Midulla F, Di Mattia G, Merven M, Janson JT (2021) Pediatric bronchoscopy: recent advances and clinical challenges. Expert Rev Respir Med 15(4):453–475. 10.1080/17476348.2021.1882854 33512252 10.1080/17476348.2021.1882854

[7] Marchant JM, Chang AB, Kennedy E, King D, Perret JL, Schultz A et al (2024) Cough in children and adults: diagnosis, assessment and management (CICADA). Summary of an updated position statement on chronic cough in Australia. Med J 220(1):35–45. 10.5694/mja2.52157. Epub 2023 Nov 19. Erratum in: Med J Aust 2024 Jul 15;221(2):91. 10.5694/mja2.52363 10.5694/mja2.52157 37982357

[8] Morice AH, Millqvist E, Bieksiene K, Birring SS, Dicpinigaitis PV, Ribas CD et al (2020) ERS guidelines on the diagnosis and treatment of chronic cough in adults and children. Eur Respir J 55:1901136. 10.1183/13993003.01136-2019 31515408 10.1183/13993003.01136-2019 PMC6942543

[9] Faniran AO, Peat JK, Woolcock AJ (1998) Persistent cough: is it asthma? Arch Dis Child 79:411–414. 10.1136/adc.79.5.411 10193253 10.1136/adc.79.5.411 PMC1717737

[10] Röder M, Ng AYKC, Conway MA (2024) Bronchoscopic diagnosis of severe respiratory infections. J Clin Med 13(19):6020. 10.3390/jcm13196020 39408080 10.3390/jcm13196020 PMC11477651

[11] Bergamini M, Kantar A, Cutrera R, Interest Group IPC (2017) Analysis of the literature on chronic cough in children. Open Respir Med J 27(11):1–9. 10.2174/1874306401711010001 10.2174/1874306401711010001 PMC5427690 28553418

[12] Chang AB, Landau LI, Van Asperen PP, Glasgow NJ, Robertson CF, Marchant JM, Mellis CM, Thoracic Society of Australia and New Zealand (2006) Cough in children: definitions and clinical evaluation. Med J 184(8):398–403. 10.5694/j.1326-5377.2006.tb00290.x 10.5694/j.1326-5377.2006.tb00290.x 16618239

[13] Terkawi RS, Altirkawi KA, Terkawi AS, Mukhtar G, Al-Shamrani A (2016) Flexible bronchoscopy in children: utility and complications. Int J Pediatr Adolesc Med 3(1):18–27. 10.1016/j.ijpam.2015.12.003 30805463 10.1016/j.ijpam.2015.12.003 PMC6372410

[14] Gallucci M, Pedretti M, Giannetti A, di Palmo E, Bertelli L, Pession A, Ricci G (2020) When the cough does not improve: a review on protracted bacterial bronchitis in children. Front Pediatr 7(8):433. 10.3389/fped.2020.00433 10.3389/fped.2020.00433 PMC7426454 32850546

[15] Song WJ, An J, McGarvey L (2020) Recent progress in the management of chronic cough. Korean J Intern Med 35(4):811–822. 10.3904/kjim.2020.013 32422697 10.3904/kjim.2020.013 PMC7373968

[16] Cheng ZR, Chua YX, How CH, Tan YH (2021) Approach to chronic cough in children. Singapore Med J 62(10):513–519. 10.11622/smedj.2021200 35001125 10.11622/smedj.2021200 PMC8804402

[17] Weinberger M, Hurvitz M (2020) Diagnosis and management of chronic cough: similarities and differences between children and adults. F1000Res 22;9:F1000 Faculty Rev-757. 10.12688/f1000research.25468.1 10.12688/f1000research.25468.1 PMC7385707 32765833

[18] Bush A (2017) Persistent bacterial bronchitis: time to venture beyond the umbrella. Front Pediatr 5:264. 10.3389/fped.2017.00264 29322037 10.3389/fped.2017.00264 PMC5732151

[19] Goussard P, Gie R (2014) The role of bronchoscopy in the diagnosis and management of pediatric pulmonary tuberculosis. Expert Rev Respir Med 8(1):101–109. 10.1586/17476348.2013.863712 24378192 10.1586/17476348.2013.863712

[20] Nicolai T (2001) Pediatric bronchoscopy. Pediatr Pulmonol 31:150–164. 10.1002/ppul.1024 11180692 10.1002/1099-0496(200102)31:2<150::aid-ppul1024>3.0.co;2-6

[21] Pan W, Peng D, Luo J, Liu E, Luo Z, Dai J, Fu Z, Li Q, Huang Y (2014) Clinical features of airway malacia in children: a retrospective analysis of 459 patients. Int J Clin Exp Med 7(9):3005–3012 25356175 PMC4211825

[22] Kamran A, Zendejas B, Jennings RW (2021) Current concepts in tracheobronchomalacia: diagnosis and treatment. Semin Pediatr Surg 30(3):151062. 10.1016/j.sempedsurg.2021.151062 34172207 10.1016/j.sempedsurg.2021.151062

[23] Chung KF, McGarvey L, Song WJ, Chang AB, Lai K, Canning BJ, Birring SS, Smith JA, Mazzone SB (2022) Cough hypersensitivity and chronic cough. Nat Rev Dis Primers 8:45. 10.1038/s41572-022-00370-w 35773287 10.1038/s41572-022-00370-w PMC9244241

[24] Heching M, Rosengarten D, Shitenberg D, Yigla M (2020) Bronchoscopy for chronic unexplained cough: use of biopsies and cultures increase diagnostic yield. J Bronchology Interv Pulmonol 27:30–35. 10.1097/LBR.0000000000000629 31651543 10.1097/LBR.0000000000000629

